# Microevolution of *Campylobacter jejuni* during long-term infection in an immunocompromised host

**DOI:** 10.1038/s41598-020-66771-7

**Published:** 2020-06-22

**Authors:** Clare R. Barker, Anaïs Painset, Craig Swift, Claire Jenkins, Gauri Godbole, Martin C. J. Maiden, Timothy J. Dallman

**Affiliations:** 1 Department of Zoology, Peter Medawar Building, University of Oxford, Oxford, UK; 2 Gastrointestinal Bacteria Reference Unit, Public Health England, Colindale London, UK; 3 NIHR Health Protection Research Unit in Gastrointestinal Infections, Liverpool, UK; 4 Division of Infection and Immunity, The Roslin Institute and Royal (Dick) School of Veterinary Studies, University of Edinburgh, Edinburgh, UK

**Keywords:** Bacteriology, Immunological disorders

## Abstract

Campylobacteriosis typically manifests as a short-lived, self-limiting gastrointestinal infection in humans, however prolonged infection can be seen in cases with underlying immunodeficiency. Public Health England received 25 isolates of *Campylobacter jejuni* from an individual with combined variable immunodeficiency over a period of 15 years. All isolates were typed and archived at the time of receipt. Whole genome sequencing (WGS) and antimicrobial susceptibility testing were performed to examine the relatedness of the isolates and to investigate the changes in the genome that had taken place over the course of the infection. Genomic typing methods were compared to conventional phenotypic methods, and revealed that the infection was caused by a single, persistent strain of *C. jejuni* belonging to clonal complex ST-45, with evidence of adaptation and selection in the genome over the course of the infection. Genomic analysis of sequence variants associated with antimicrobial resistance identified the genetic background behind rRNA gene mutations causing variable levels of resistance to erythromycin. This application of WGS to examine a persistent case of campylobacteriosis provides insight into the mutations and selective pressures occurring over the course of an infection, some of which have important clinical relevance.

## Introduction

*Campylobacter* species, principally *Campylobacter jejuni* (~90% of cases) and *Campylobacter coli* (~10% of cases), are the most common cause of bacterial gastroenteritis in the United Kingdom^1^. Human infection is largely acquired through consumption or handling of contaminated poultry meat^2^. Campylobacteriosis in otherwise healthy persons is a self-limiting disease, which typically lasts less than ten days and rarely requires antibiotic therapy. In immunocompromised cases the disease can however be more severe and despite treatment, may persist for long periods of time.

The immune response to *Campylobacter* infection is mediated by a combination of adaptive and innate responses^3^, with defects in these processes, such as primary immunodeficiencies or Human Immunodeficiency Virus (HIV) infection, linked to more severe or prolonged disease^4^. As well as an increased likelihood of serious complications including bacteraemia, these patients may also develop recurrent infections^5^ and sometimes chronic carriage of *Campylobacter* despite antibiotic treatment. Reported cases of chronic infection with *Campylobacter* in patients with hypogammaglobulinaemia, an immune disorder resulting in a reduction of gamma globulins, range from several months^6–8^ to six years^9^ with additional reports of potential cases lasting for 17 years^10^ and 25 years^11^. The return of a *C. jejuni* infection following antibiotic treatment in an immunocompetent patient has been described^12^. The majority of these long-term studies of *Campylobacter* carriage have not ascertained whether these cases are due to an initial infection, and subsequent colonisation with a single population of bacteria, or if they are the result of multiple, repeated infections. A PCR-based method determined that a 16-month infection in a patient with hypogammaglobulinaemia was the result of two strains of *C. jejuni*: a transient strain and a persistent strain^13^. The authors of this study concluded that their method was not suitable for examining clonal patterns over extended periods of time. Utilising whole genome sequencing (WGS) technology, it is possible to examine the relatedness of isolates to single nucleotide resolution and therefore track changes in the bacterial genomes over the course of the host’s infection. This has been highlighted by a study which examined the colonisation of a human host by a single strain of *C. jejuni*, categorising its evolution and adaptation^14^.

Within-host population genetics and microevolution are important emerging areas of microbiology, which are accessible by WGS studies, and there have been several reports examining the evolution of single clones of bacteria within the same host over several years^14–19^. Bacterial infections in immunocompromised patients are particularly interesting as they present an environment with altered immune selection, and allow long-term growth in a stable setting. At the time of writing, study of long-term within-host microevolution in immunocompromised patients had been examined in very few bacterial species^14,20^. Much of the difficulty lies in gaining access to a viable collection of patient isolates that have been gathered over a long period of time.

The Gastrointestinal Bacteria Reference Unit (GBRU) at Public Health England (PHE) receives human isolates of *Campylobacter* spp. for species identification, typing and antimicrobial susceptibility testing. All isolates received are archived in frozen storage, providing a unique opportunity to examine isolates that are relevant to public health and which have been collected over time. Here, WGS was applied to a collection of 25 *Campylobacter jejuni* isolates received by GBRU over the course of a 15-year infection in a case with combined variable immunodeficiency (CVID). The relatedness of these isolates, their population structure and analyses of the changes that have occurred over time, particularly in relation to antibiotic resistance, are described.

## Results

### Sequence typing

**Figure 1:**
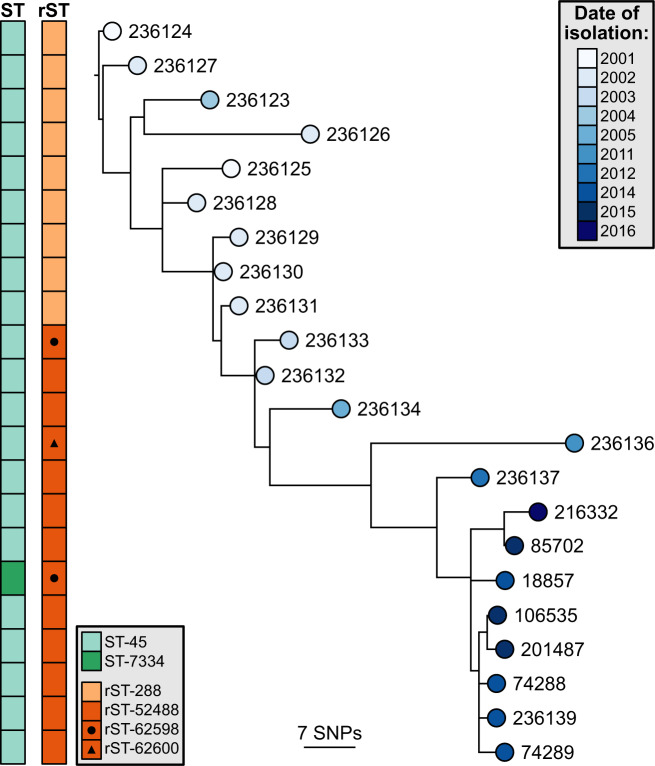
Phylogenetic analysis of the isolates revealed a ladder-like tree. SNP-based maximum-likelihood phylogeny of the core genome, rooted on the oldest isolate. The tips are coloured according to the date of isolation, and sequence typing results are displayed to the left. ST, sequence type; rST, ribosomal sequence type; SNP, single nucleotide polymorphism.

To determine the genetic relatedness between the isolates, sequence typing was performed at three levels of resolution. 7-gene MLST revealed that all but one of the isolates belong to ST-45, except isolate 18857 (ST-7334), which differed by a single nucleotide in *uncA* (Fig. 1). All isolates however belonged to the ST-45 clonal complex. Ribosomal sequence typing (rMLST), which targets the 52 genes that encode ribosomal proteins in *Campylobacter*^21,22^, identified variation at three loci: *rpsA*, *rpsJ*, and *rpsR* – encoding the 30 S ribosomal proteins S1, S10 and S18 respectively (Supplementary Table S1). The mutation in *rpsJ* was identified in isolates 236133 and 18857, and the mutation in *rpsR* was identified in isolate 236136. A 3-base TAG insertion in *rpsA* was initially identified in isolate 236133 and was subsequently detected in every isolate thereafter (Fig. 1, Supplementary Table S1). Variation was also quantified across the 1,343 loci defined by the core genome sequence typing (cgMLST) *C. jejuni* and *C. coli* scheme^23^. The isolates within this study were found to vary at 104 (7.7%) of these loci, which included the three variable rMLST loci (Supplementary Table S2). Isolate 18857 that was a single locus variant by MLST was subsequently identified to vary by only 4 cgMLST loci from the next closest isolate, excluding incomplete loci.

### Root-to-tip & temporal analyses

**Figure 2:**
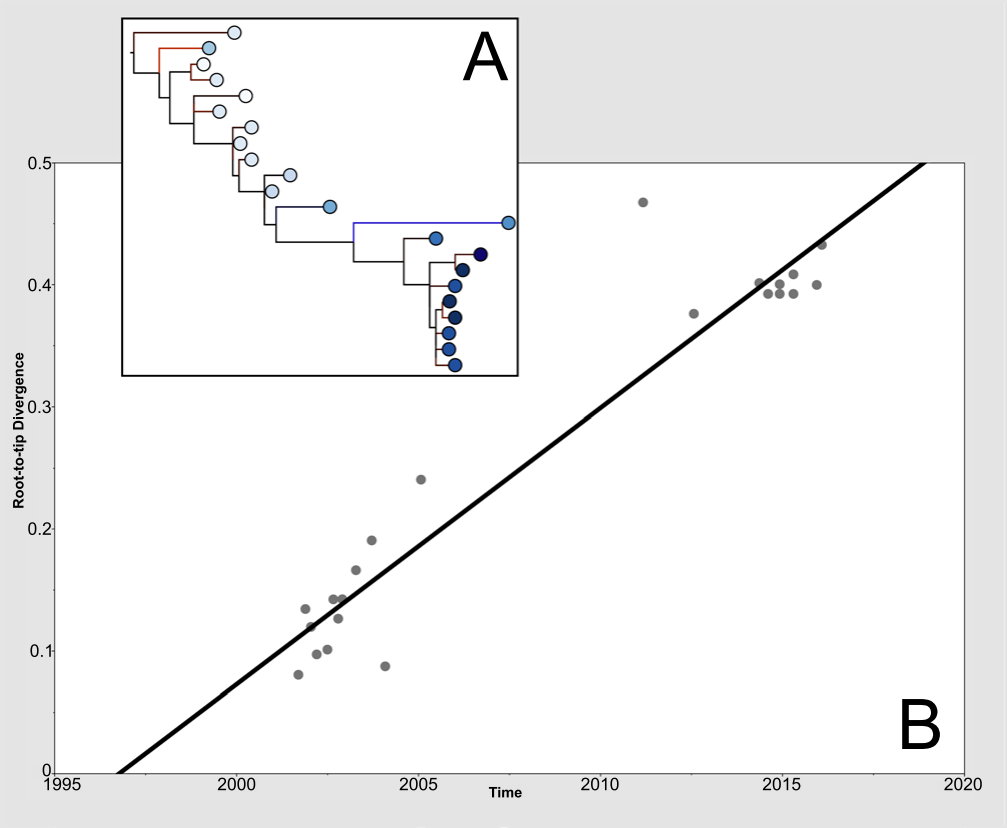
Root-to-tip analysis identified a temporal signal in the genomic data. (**A**) Phylogenetic tree with the best-fitting root, as determined by TempEst software^61^, tips coloured according to date of isolation. (**B**) Root-to-tip linear regression plot showing date of isolation versus branch length. Slope = 2.26e^−2^, TMRCA = 1996.75, R^2^ = 0.915, correlation coefficient = 0.957. Blue branches represent points below the regression line, indicating sequences that are less divergent (for their sampling date) than average. Red branches represent the opposite situation.

The population structure of the isolates was investigated phylogenetically based on nucleotide variation across a core genome alignment (Fig. 1). A root-to-tip regression analysis was performed to assess the presence of a temporal signal (Fig. 2). A strong correlation was observed between isolation date and branch length (R^2^ = 0.915, correlation coefficient = 0.967) consistent with a constant accrual of diversity over the course of infection. The best-fitting root was isolate 236126 (January 2002) and the estimated time to most recent common ancestor was October 1996. The mutation rate was estimated to be 1.75e^−6^ substitutions per site per year. A similar rate of mutation based on a genome size of 1,641,481 bp^24^ was determined using Bayesian temporal analysis: 2.07e^−6^ subs per site per year, 95% CI = 1.56e^−6^–2.59e^−6^.

### Microevolution during colonisation

**Figure 3:**
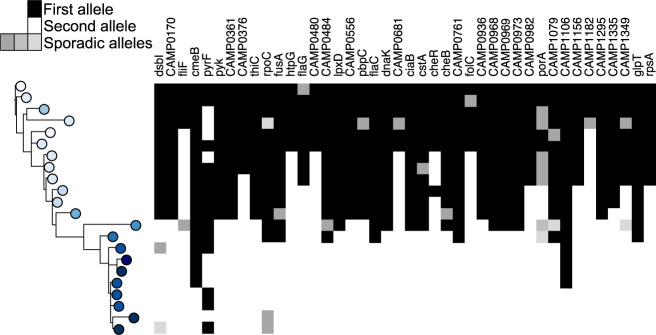
Acquisition and fixation of mutations over time in 42 loci. Tree topology and colouring as in Fig. 1.

Of the 104/1,343 cgMLST loci that were found to be variable within the patient’s isolates, 62 (59.6%) were sporadic, with an allele change occurring in three or fewer isolates with no obvious inheritance (Supplementary Table S2). In the remaining 42 loci, alleles occurred and became fixed after different time points, appearing in the majority of isolates after that time (Fig. 3). Of these 42 loci, 15 (35.7%) also had sporadic mutations (Fig. 3, Supplementary Table S2).

The 42 loci at which this ‘fixed’ typed of allelic variation occurred were investigated further; over three-quarters (83%) of these had accumulated non-synonymous mutations. In 11/42 alleles (26.2%) a frameshift had occurred due to an indel, which were also identified in 23 (37.1%) of the alleles containing sporadic mutations. In total, almost two-thirds (31/104, 29.8%) of all the variable core loci were affected by frameshift mutations over the course of the infection.

### Comparison with previous isolates

**Figure 4:**
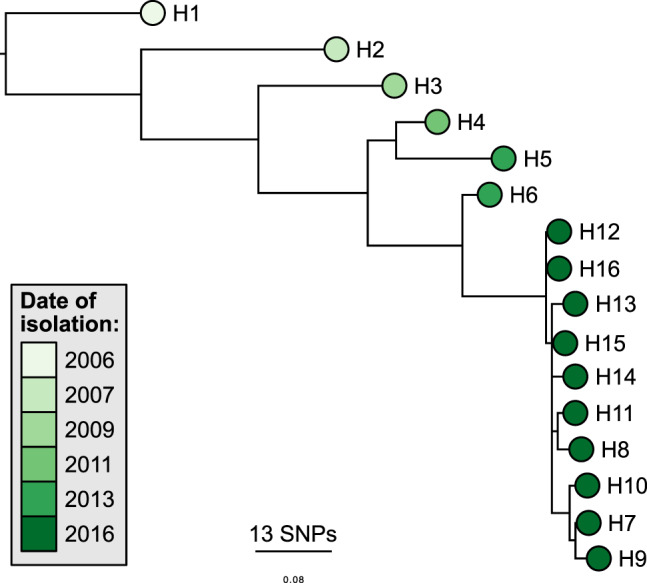
Phylogenetic analysis of the comparison isolates^14^ revealed a similar, ladder-like tree. SNP-based maximum-likelihood phylogeny of the core genome, rooted on the oldest isolate. The tips are coloured according to the date of isolation. SNP, single nucleotide polymorphism.

A previously published study also investigated the adaptation of a single clone of *C. jejuni* ST-45 in an immunocompromised patient^14^. We downloaded the genomes from this study and performed our workflow of sequence typing, temporal analysis and microevolution analysis upon these isolates, to investigate the reproducibility of the results shown here. The results showed a similarly-shaped phylogeny (Fig. 4), root-to-tip linear regression data (slope = 2.96e^−2^, R^2^ = 0.9801, correlation coefficient = 0.99), and mutation rate (5.55e^−6^). Additionally, there were a similar number of fixed mutations (33 loci), whilst not the same loci as identified in this study many also occurred in genes encoding cell surface structures such as capsular polysaccharide proteins and flagellae, as well as chemotaxis proteins (Supplementary Table S3).

### Antibiotic resistance

All isolates displayed resistance to ciprofloxacin and nalidixic acid, with MIC values ranging from 4 mg/l to>32 mg/l for ciprofloxacin and from 64 mg/l to>512 mg/l for nalidixic acid (Supplementary Table S4). The phenotypic resistance to fluoroquinolones was conferred by the presence of two mutations in the quinolone resistance determining region (QRDR) of *gyrA* (257 C > T and 268 G > A) resulting in amino acid substitutions Thr86Ile and Asp90Asn.

**Figure 5:**
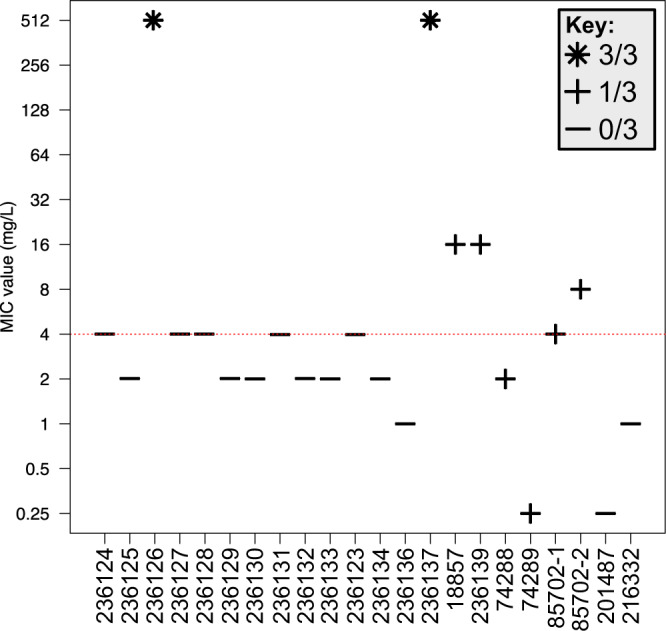
Erythromycin MIC values of isolates and corresponding 23 S rRNA gene mutations. Red line represents the EUCAST breakpoint value. Icons identify the number of copies of the 23 S rRNA gene that have acquired a mutation in each isolate. MIC, minimum inhibitory concentration.

Phenotypic resistance to erythromycin was variable over the course of the infection (Fig. 5). The first four isolates received (September 2001 – March 2002) were susceptible, until 236126 (July 2002) which exhibited a high level of resistance (MIC > 512 mg/l). Resistance in this isolate was caused by a 2075 A > G transition in each of the three copies of the 23 S rRNA gene (Fig. 5, Table 1). Subsequent isolates did not carry any 23 S mutation and were phenotypically susceptible until July 2012, when isolate 236137 exhibited high level resistance (MIC > 512 mg/l). For this isolate the resistance was conferred by a 2074 A > C transversion in all three copies of the 23 S rRNA gene. Subsequent isolates however, only carried the 2074 A > C mutation in one of the three copies. The single mutated copy of the 23 S rRNA gene was maintained until April 2015, and the MIC values between these dates varied between 0.25 mg/l and 16 mg/l.

**Table 1:** Reads covering the 23 S rRNA gene, mutations at position 2074 and corresponding phenotype.

Isolate	Number of reads covering position 2074	Number of reads mapping to A	Percent of reads mapping to A	Mutation (if present)	23 S rRNA copies mutated	MIC (mg/l)
236124	1,359	1,348	99.19%	—		4
236125	2,310	2,284	98.87%	—		2
236126^a^	1,592	5	0.31%	99.37% A > G	3	512*
236127	1,749	1,739	99.43%	—		4
236128	1,776	1,767	99.49%	—		2
236129	1,555	1,547	99.49%	—		2
236130	1,913	1,896	99.11%	—		4
236131	1,529	1,520	99.41%	—		2
236132	1,760	1,752	99.55%	—		4
236133	1,924	1,902	98.86%	—		2
236123	1,267	1,261	99.53%	—		4
236134	2,913	2,899	99.52%	—		2
236136	2,081	2,068	99.38%	—		1
236137	1,542	8	0.52%	99.42% A > C	3	512*
18857	591	390	65.99%	32.99% A > C	1	16*
236139	2,097	1,366	65.14%	34.29% A > C	1	16*
56946^b^	1,743	1,167	66.95%	32.99% A > C	1	—
74288	2,268	1,606	70.81%	29.10% A > C	1	2
74289	2,367	1,488	62.86%	37.14% A > C	1	0.25
85702	1,024	648	63.28%	36.52% A > C	1	4
						8*
106535^b^	1,349	926	68.64%	31.13% A > C	1	—
	1,827	1,131	61.90%	38.04% A > C	1	—
201487	2,258	2,256	99.91%	—		0.25
216332	1,774	1,770	99.77%	—		1

Isolates U1 and U2 could not be regrown from frozen storage therefore no genotype was available for these isolates. ^a^The results are for position 2075. ^b^Isolates were sequenced upon receipt but could not be regrown from frozen storage and so genotypic but not phenotypic results were available for these isolates. Asterisks indicate MIC values corresponding with resistance to erythromycin. MIC, minimum inhibitory concentration.

## Discussion

While there exist numerous studies examining how bacteria vary over time at the population level, there are relatively few on how bacteria differ within a single host throughout an infection presumably due to the often transient nature of colonisation during infection and relatively slow mutation rate of bacteria.. Consequently, within-host studies of bacteria typically involve chronic pathogens such as *Mycobacterium tuberculosis*^18,25,26^ and *Helicobacter pylori*^15,16^. Immunodeficient patients with persistent infections can provide an opportune environment for studying the evolution of bacteria with normally shorter infection cycles. Here, we have examined the microevolution that occurred in a single clone over the course of a fifteen-year case of campylobacteriosis; where the pathogen in question is typically cleared within two weeks. By performing genomic analyses on this collection of serially sampled isolates we have gained insight into how this population of *C. jejuni* has evolved over the course of infection in an environment with reduced immune selection.

The isolates obtained are closely related and form a single population consistent with a single founding strain effectively colonising the gut of the patient. This finding suggests that serotyping is not a robust marker of clonality in *Campylobacter*. Phylogenetic analysis identified a strong temporal signal with respect to the accumulation of mutations. The mutation rate of this population was estimated to be 2.07e^−6^ per site, which approximates to 3.5 mutations in the genome each year. The SNP variation across the isolates suggests that, whilst there is a single dominant strain persisting within this individual, there were sub-clones being sampled at the different time points, leading to the multiple sub-clades within the reconstituted phylogeny. This dominant strain can be seen in the backbone of all the phylogenetic trees (Figs. 1–3), which are ladder-like in nature.

A six year gap between samples was observed between 2005 and 2011where no isolates were sent to the reference laboratory for typing from this patient. We are unable to say if the patient was symptomatic or not during this period or if isolates were not sent for another reason (*Campylobacter* isolates are sent to the reference laboratory voluntarily from diagnostic laboratories in the UK). Whilst we cannot unequivocally state this is a single infection throughout the time series as opposed to a secondary infection by a genetically similar strain we feel the former is the most plausible explanation based on the genome sequences for the following reasons (a) The level of relatedness between 2001–2005 and 2011–2016 strains is consistent with a steady accrual of variation in-line with the strong regression seen in root-to-tip divergence (b) The fact that the 2011–2016 diversity is descendant of the diversity seen in 2001–2005 supports a continual evolution of a strain in a closed system.

To examine the genes that have changed over the course of the infection the 1,343 loci within the *C. jejuni* cgMLST scheme were examined. Of those that were variable, over one third were present in all subsequent isolates and over half were non-synonymous changes, and so plausibly sites under selection. Despite the immunocompromised status of the patient, many of the proteins encoded by these loci are located on the bacterial cell surface, consistent with selection caused by the host immune system. These indications of host-pathogen selection and the phylogenetic laddering, hallmark of directional selection, suggest this population may have been under strong diversifying selection from host responses. Given the immunocompromised status of the patient, other evolutionary mechanisms, such as population bottlenecks, may also be responsible for the changes seen during the infection. It has been proposed that in situations where immune selection is absent, non-selective population dynamic process such as genetic bottlenecks could produce temporal clustering in a phylogenetic tree^27,28^. It is likely that any such bottleneck was small, leading to a shift in the prevalence of certain variants, as larger bottleneck sizes in *C. jejuni* have been shown to maintain population diversity^29^. This potential bottleneck may have been caused by any number of reasons, including: antibiotic or immunotherapy treatment; competition from the surrounding microbiota or a local co-infection; or a change in environmental conditions such as reduced nutrients.

Over one quarter of the fixed non-synonymous mutations were frameshift mutations caused by indels, alongside more than one third of the sporadic mutations. As the genome of *C. jejuni* is known to contain ‘at least twenty’ pseudogenes^24^, the formation of an additional thirty pseudogenes over fifteen years is notable. Based on the 1,343 loci in the cgMLST scheme of *C. jejuni*^23^, these 31 pseudogenes translate to ~2.3% of the core protein coding capacity. This is far lower than the 300 genes (~6.9%) reported in a study which investigated a 15-year *Salmonella enterica* Enteritidis infection in an immunocompromised patient^20^. In their case however, the causative organism was a *mutS* mutant, with a possible 1,000-fold increased rate of frameshift mutations. Considering the different protein encoding capacities of the two species (1,343 versus 4,347 genes) as well as the increased frameshift rate of the *S*. Enteritidis isolates, this is consistent with a deletional bias in the *C. jejuni* isolates. The deletional bias may be the result of a lack of selection, which would otherwise be driven by the immune system, or ineffective selection due to a small population size. A small population size is consistent with there being repeated population bottlenecks, as was suggested by the phylogenetic data. The high frequency of pseudogene formation resulting from the lack of selective pressure against deletional mutations may additionally be evidence of host adaptation and genome degradation occurring on a reduced timescale, as was observed by Klemm *et al*.^20^.

A similar study looking at the genomics of long-term campylobacteriosis in an immunocompromised patient also, interestingly, involved a strain of ST-45 *C. jejuni*^14^. We compared the rate and type of microevolutionary events in this dataset of isolates with those from this study. The results confirm our conclusions; that there is a strong molecular clock signal and that within-host microevolution of *C. jejuni* leads to non-synonymous mutations that become fixed within the population. many of which may affect cell surface proteins. The analysis of this additional dataset also supports our findings on the calculated mutation rate of *C. jejuni*, and correlates well with the estimated value by Wilson *et al*.^30^. Additionally, other studies investigating isolates from single patients have also found mutations in similar genes, such as the motility accessory factor family and chemotaxis proteins^22,31^, lending support to the hypothesis that this is a regular occurrence within *C. jejuni* genomes during infection.

Previous studies of the passage of *C. jejuni* through animal and human hosts have revealed the occurrence of mutations in homopolymeric tracts of contingency loci, with frameshift mutations in genes regulating surface structure biosynthesis^32,33^. While we detected multiple alleles in eleven of the contingency loci detailed by Jerome *et al*.^32^ (Supplementary Table S5), these were all sporadic mutations and did not appear to become fixed within the population, but reinforced that these loci are mutation hotspots.

Antibiotic resistance in *Campylobacter* is a major clinical and public health concern, with the World Health Organisation adding fluoroquinolone-resistant *Campylobacter* to its 2017 global priority list of antibiotic resistant pathogens deemed to pose the greatest threat to human health^34^. In cases where treatment is required, such as in immunocompromised patients where infection is likely to be more severe, the two most commonly prescribed antibiotics for campylobacteriosis are erythromycin and ciprofloxacin. Within this collection of isolates, we found resistance to both antibiotics, with phenotypic data supporting the presence of previously characterised single point mutations.

Quinolone resistance has been described in *C. jejuni* for over 25 years, arising shortly after the introduction of fluoroquinolones for veterinary use^35,36^ and increasing in prevalence over time^37^. It is unusual that two mutations (Thr86Ile and Asp90Asn) are present in every isolate in this study, as most fluoroquinolone resistance in *C. jejuni* is the result of a single mutation in *gyrA*^38–41^. As previously described, the Thr86Ile mutation is associated with high level resistance (≥16 mg/l) to ciprofloxacin, lower levels of resistance are caused by the Asp90Asn mutation (≤8 mg/l), and the presence of both results in the highest level of resistance (≥128 mg/l)^42,43^. This high-level, stable resistance to one of the first-line campylobacteriosis treatments is of concern, especially considering the immune status of the patient, and may have contributed to the initial persistence of the bacterial population.

The erythromycin resistance of these isolates was dynamic: all cases of resistance were shown to be caused by non-synonymous point mutations in the peptidyl-encoding region of domain V of the 23 S rRNA gene, of which *C. jejuni* carries three copies^24^. Initially, high level resistance appeared once in isolate 236126 in January 2002, the result of a 2075 A > G mutation in three gene copies. This mutation, the most commonly found in *Campylobacter*^44,45^, is known to produce high level, stable resistance^44–46^, but was not maintained in this population. The isolates remain sensitive until a 2074 A > C mutation arises sometime between March 2011 and July 2012. This mutation is present in all three copies of the 23 S rRNA gene and leads to high-level resistance to erythromycin, again consistent with previous literature^47^. The mutation was not observed in two of the three gene copies at some point between July 2012 and May 2014, resulting in variable levels of resistance in subsequent isolates. Although information on the specific antibiotic regime of the patient referred to in this study was not available, it is possible that this change in resistance genotype could be a result of cessation of macrolide treatment leading to a lack of selective pressure, or, perhaps more likely, instead be due to the fitness cost that is exerted on *C. jejuni* strains carrying mutations in the 23 S rRNA gene, as observed by other studies^47,48^.

The lower MIC values seen in those isolates with a single mutated copy of the 23 S rRNA gene were consistent with previous findings that fewer than three mutations lead to lower MIC values^45,49^. Both studies also report that macrolide resistance was not found at all in *Campylobacter* isolates with only one mutated copy of the gene; however, our findings show that low level resistance to erythromycin is possible when just a single copy of the gene carries a mutation in the macrolide binding site. This effect appears to be variable, perhaps due to compensation by the remaining two wild-type ribosomal genes. It is also possible that the mutation is present unevenly within the bacterial population, or is rapidly lost during culture due to fitness costs of maintaining the polymorphism, and therefore different results are produced depending upon the colony chosen for phenotypic testing. Isolate 85702, which had two separate colonies archived at the time of receipt, provides evidence towards this theory, as one was found to be resistant and the other susceptible, despite deriving from the same sample. Additionally, isolates 74288 and 74289, which were collected on the same day, show a large difference in their MIC values. The presence of the single mutation, while not necessarily leading to resistance, may be a way for the bacteria to maintain the resistance mutation whilst negating the fitness costs associated with multiple copies of the mutation, making it easier to gain high-level, stable resistance by accumulating further mutations.

## Conclusion

This study is among the first to examine the microevolution of a long-term campylobacteriosis infection in an immunocompromised human host. This unique situation – a natural experiment involving the colonisation of a single clone followed by evolution, with no subsequent infections and no lateral gene transfer – allowed observation of evolutionary processes that may otherwise occur on a far greater timescale, and an insight into the potential beginnings of host adaptation and genome degradation. The contemporaneous sequencing and analysis of isolates from chronic infections is an important step forward in public health microbiology. By tracking the evolution of a strain, we can potentially inform clinical decisions such as antibiotic treatment in real-time, when mutations arise, and ultimately aim to reduce the length of disease in cases of chronic infection such as the one presented here.

## Methods

### Bacterial isolates

Public Health England (PHE) received a total of 25 *C. jejuni* isolates (Table 2) over the course of 15 years, derived from single colonies from stool samples belonging to a patient with CVID with symptoms of persistent diarrhoea. From 2001 until 2015, these isolates were typed using serotyping and/or phage typing. In 2015, 7-gene multilocus sequence typing (MLST)^50,51^ was introduced at PHE, and from January 2015 onwards the isolates underwent routine whole genome sequencing (WGS) upon receipt. For the purpose of this study, all viable isolates from the archive were genome sequenced (n = 23). Isolates were retrieved from −80 °C storage on beads and regrown on Columbia Blood Agar (CBA) media at 37 °C under microaerobic conditions for 48–72 hours.

**Table 2:** Summary of 25 *C. jejuni* isolates received over 15 year period from this patient; information provided by routine method of testing in use at time of receipt.

Isolate	Isolation Date	HS	PT	ST	AST	WGS
236124	2001-09	37	2	45	✓	✓
236125	2001-11	Untypable	2	45	✓	✓
236126	2002-01	Untypable	2	45	✓	✓
236127	2002-03	12	2	45	✓	✓
236128	2002-07	13	2	45	✓	✓
236129	2002-09	13	2	45	✓	✓
236130	2002-10	13	2	45	✓	✓
236131	2002-11	13	2	45	✓	✓
236132	2003-04	13	2	45	✓	✓
236133	2003-09	Untypable	2	45	✓	✓
236123	2004-02	37	2	45	✓	✓
236134	2005-01	12	2	45	✓	✓
236136	2011-03	12	2	45	✓	✓
U1	2011-04	10/12	2	×^b^	×^b^	×^b^
236137	2012-07	12	2	45	✓	✓
U2	2014-05	ND	2	×^b^	×^b^	×^b^
18857	2014-05	ND	2	7334	✓	✓
236139	2014-08	ND	2	45	✓	✓
56946	2014-09	ND	ND	45	×^c^	✓^d^
74288	2014-12	ND	ND	45	✓	✓
74289	2014-12	ND	ND	45	✓	✓
85702	2015-01	ND	ND	45	✓✓^e^	✓
106535	2015-04	ND	ND	45	×^c^	✓
201487	2015-12	ND	ND	45	✓	✓
216332	2016-01	ND	ND	45	✓	✓

^a^Date of isolation unknown; shown instead is the date the isolate was received. ^b^No sequencing data available. ^c^Originally sequenced upon receipt but could not be revived for the enhanced antimicrobial susceptibility testing. ^d^Contaminated genome sequence, isolate not included in genomic analysis. ^e^Antimicrobial Susceptibility Testing (AST) performed twice on separate archived copies of isolate. Isolate 106535 has limited information on AST results from the original testing. Heat-stable antigen, serotype; HS, Penner serotype; PT, phage type; ST, sequence type; AST, antimicrobial susceptibility testing; WGS, whole genome sequencing; ND, not done.

Two isolates could not be regrown and so WGS data and repeat antimicrobial susceptibility results are not available for these isolates. Isolate 18857 was received on the same day as isolate U2. Isolates 74288 and 74289 were also both received on the same day. Isolate 85702 was archived twice. Two of the previously sequenced isolates from 2014 and 2015 (56946 and 106535 respectively) could not be regrown and therefore were not available for phenotypic antimicrobial susceptibility testing.

### Antimicrobial susceptibility testing

Antimicrobial susceptibility was determined using agar dilution methodology and interpreted according to the European Committee on Antimicrobial Susceptibility Testing (EUCAST) clinical breakpoints or epidemiological cut-offs^52^ (Supplementary Table S6). Sixteen of the isolates were originally tested for antimicrobial susceptibility upon receipt, though the panel of antimicrobials varied over the years. Isolates U1 and 106535, which could not be regrown, were both described in the laboratory database as being resistant to ciprofloxacin upon initial receipt, although the minimum inhibitory concentration (MIC) value was not noted.

### DNA extraction and whole genome sequencing

Following overnight culture on CBA media, isolates were inoculated into Brain Heart Infusion broth and lysed using tissue lysis buffer, proteinase K and RNase A (Qiagen QIAsymphony DNA Mini Kit). Genomic DNA from the cell lysates was extracted using a QIAsymphony automated DNA extraction system (Qiagen). Libraries were prepared using the Illumina Nextera XT kit and sequenced using an Illumina HiSeq. 2500 in rapid run mode, producing 100 bp paired-end reads (Supplementary Table S7).

### Bioinformatic analyses

The PHE bioinformatics pipeline characterises sequence types using MOST^53^ with allelic profiles derived from the PubMLST *Campylobacter* database^54^. Antimicrobial resistance determinants are identified by mapping reads to known resistance genes, as described in Swift *et al*.^55^, and genome assembly is performed using SPAdes^56^ (assembly statistics available in Supplementary Table S7). Parsnp from the Harvest software suite^57^ was used to generate core genome SNP alignments and subsequent phylogenies using FastTree 2^58^ from the assembled genomes, and Snippy^59^ was used to match mutations to reference genes. A check for recombination was performed using Gubbins^60^. Root-to-tip linear regression was performed using TempEst software^61^. Ribosomal^21^ and core genome MLST^23^ analyses were performed using the Genome Comparator function of BIGSdb^54,62^, and the PubMLST *Campylobacter* database was queried to identify closely related isolates. Core genome SNP alignments produced by Parsnp were analysed using the BEAST v.2.4.4 package^63^ to estimate mutation rates, with tip dates specified as each isolate’s year of isolation. Following model testing using jModelTest2^64^ the HKY model of nucleotide substitution with equal base frequencies in order to simulate the K80 model was identified as the best substitution model for these data. A strict molecular clock as per the results of the root-to-tip linear regression was selected, starting with an estimated clock.rate of 2.92e^−6^, using the coalescent constant population model. Five independent Markov Chain Monte Carlo (MCMC) chains were run, each with a 1 million burn-in and 9 million chain length, sampled every 10,000 states.

### Ethics approval

This study was exempted from requiring ethical approval following internal review by Public Health England.

## Supplementary information

Supplementary tables.

## Data Availability

The sequences produced during this study are available in the NCBI Sequence Read Archive repository under BioProject PRJNA505131. The individual accession numbers are provided in Supplementary Table S7. Additionally, all genomes are available in the PubMLST *Campylobacter* database (available at: pubmlst.org/campylobacter), with PubMLST ID numbers also provided in Supplementary Table S7.

## References

[1] Tam CC (2012). Changes in causes of acute gastroenteritis in the United Kingdom over 15 years: Microbiologic findings from 2 prospective, population-based studies of infectious intestinal disease. Clinical Infectious Diseases.

[2] Sheppard SK (2009). *Campylobacter* Genotyping to Determine the Source of Human Infection. Clin. Infect. Dis..

[3] Janssen R (2008). Host-pathogen interactions in *Campylobacter* infections: The host perspective. Clinical Microbiology Reviews.

[4] Tee W, Mijch A (1998). *Campylobacter jejuni* Bacteremia in Human Immunodeficiency Virus (HIV)‐ Infected and Non‐HIV‐Infected Patients: Comparison of Clinical Features and Review. Clin. Infect. Dis..

[5] Le Bar WD, Menard RR, Check FE (1985). Hypogammaglobulinemia and recurrent *Campylobacter jejuni* infection. J. Infect. Dis..

[6] Pönkä A, Tilvis R, Kosunen TU (1983). Prolonged *Campylobacter* Gastroenteritis in a Patient with Hypogammaglobulinaemia. Acta Med. Scand..

[7] Green ES, Parker NE, Gellert AR, Beck ER (1984). *Campylobacter* infection mimicking Crohn’s disease in an immuno-deficient patient. Br. Med. J. (Clin. Res. Ed)..

[8] Kim Y (2017). Recurrent *Campylobacter jejuni* bacteremia in a patient with hypogammaglobulinemia: A case report. Medicine (Baltimore)..

[9] Lever AML, Dolby JM, Webster ADB, Price AB (1984). Chronic *Campylobacter* Colitis and Uveitis in Patient with Hypogammaglobulinemia. Br. Med. J..

[10] Paulet, P. & Coffernils, M. Very long term diarrhoea due to *Campylobacter jejuni*. *Postgr. Med J* **66** (1990). 10.1136/pgmj.66.775.410-a PMC2426845 2371198

[11] Ahnen DJ, Brown WR (1982). *Campylobacter* enteritis in immune-deficient patients. Ann. Intern. Med..

[12] Baqar S (2010). Recrudescent *Campylobacter jejuni* infection in an immunocompetent adult following experimental infection with a well-characterized organism. Clin. Vaccine Immunol..

[13] Moore J (2001). Investigation of infection with *Campylobacter jejuni* in a man with hypogammaglobulinaemia using PCR-single-stranded conformational polymorphism (PCR-SSCP) typing. Int. J. Med. Microbiol..

[14] Bloomfield SJ (2017). Long-term colonisation by *Campylobacter jejuni* within a human host: evolution, antimicrobial resistance and adaptation. J. Infect. Dis..

[15] Morelli G (2010). Microevolution of *Helicobacter pylori* during prolonged infection of single hosts and within families. PLoS Genet..

[16] Kennemann L (2011). *Helicobacter pylori* genome evolution during human infection. Proc. Natl. Acad. Sci. USA.

[17] Price EP (2013). Within-host evolution of *Burkholderia pseudomallei* over a twelve-year chronic carriage infection. MBio.

[18] Eldholm V (2014). Evolution of extensively drug-resistant *Mycobacterium tuberculosis* from a susceptible ancestor in a single patient. Genome Biol..

[19] Didelot X, Walker AS, Peto TE, Crook DW, Wilson DJ (2016). Within-host evolution of bacterial pathogens. Nat. Rev. Microbiol..

[20] Klemm EJ (2016). Emergence of host-adapted *Salmonella* Enteritidis through rapid evolution in an immunocompromised host. Nat. Microbiol..

[21] Jolley KA (2012). Ribosomal multilocus sequence typing: Universal characterization of bacteria from domain to strain. Microbiology.

[22] Cody AJ (2013). Real-time genomic epidemiological evaluation of human *Campylobacter* isolates by use of whole-genome multilocus sequence typing. J. Clin. Microbiol..

[23] Cody AJ, Bray JE, Jolley KA, McCarthy ND, Maiden MCJ (2017). Core genome multilocus sequence typing scheme for stable, comparative analyses of *Campylobacter jejuni* and *C. coli* human disease isolates. J. Clin. Microbiol..

[24] Parkhill J (2000). The genome sequence of the food-borne *pathogen Campylobacter jejuni* reveals hypervariable sequences. Nature.

[25] Al-Hajoj SAM (2010). Microevolution of *Mycobacterium tuberculosis* in a tuberculosis patient. J. Clin. Microbiol..

[26] Liu Q (2015). Within patient microevolution of *Mycobacterium tuberculosis* correlates with heterogeneous responses to treatment. Sci. Rep..

[27] Grenfell BT (2004). Unifying the Epidemiological and Evolutionary Dynamics of Pathogens. Science.

[28] Gray RR, Pybus OG, Salemi M (2011). Measuring the temporal structure in serially sampled phylogenies. Methods Ecol. Evol..

[29] Aidley, J., Rajopadhye, S., Akinyemi, N. M., Lango-Scholey, L. & Bayliss, C. D. Nonselective bottlenecks control the divergence and diversification of phase-variable bacterial populations. *MBio* **8** (2017). 10.1128/mBio.02311-16 PMC5380846 28377533

[30] Wilson DJ (2009). Rapid evolution and the importance of recombination to the gastroenteric pathogen *Campylobacter jejuni*. Mol. Biol. Evol..

[31] Dunn, S. J. *et al*. Genomic epidemiology of clinical *Campylobacter* spp. at a single health trust site. *Microb*. *Genomics* **4** (2018). 10.1099/mgen.0.000227 PMC6249439 30307843

[32] Jerome JP (2011). Standing genetic variation in contingency loci drives the rapid adaptation of *Campylobacter jejuni* to a novel host. Plos One.

[33] Revez J, Schott T, Llarena AK, Rossi M, Hänninen ML (2013). Genetic heterogeneity of *Campylobacter jejuni* NCTC 11168 upon human infection. Infect. Genet. Evol..

[34] Tacconelli, E. & Magrini, N. Global priority list of antibiotic-resistant bacteria to guide research, discovery, and development of new antibiotics. Available at: who.int/medicines/publications/global-priority-list-antibiotic-resistant-bacteria/en/ (2017).

[35] Endtz HP (1991). Quinolone resistance in *Campylobacter* isolated from man and poultry following the introduction of fluoroquinolones in veterinary medicine. J. Antimicrob. Chemother..

[36] Gaunt PN, Piddock LJV (1996). Ciprofloxacin resistant *Campylobacter* spp. in humans: An epidemiological and laboratory study. J. Antimicrob. Chemother..

[37] Sam WIC, Lyons MM, Waghorn DJ (1999). Increasing rates of ciprofloxacin resistant. J. Clin. Pathol..

[38] Wang Y, Huang WM, Taylor DE (1993). Cloning and nucleotide sequence of the *Campylobacter jejuni* gyrA gene and characterization of quinolone resistance mutations. Antimicrob. Agents Chemother..

[39] Ruiz J (1998). Increased resistance to quinolones in *Campylobacter jejuni*: a genetic analysis of gyrA gene mutations in quinolone-resistant clinical isolates. Microbiol. Immunol..

[40] Gibreel A, Sjogren E, Kaijser B, Wretlind B, Skold O (1998). Rapid emergence of high-level resistance to quinolones in *Campylobacter jejuni* associated with mutational changes in gyrA and parC. Antimicrob. Agents Chemother..

[41] Piddock LJV, Ricci V, Pumbwe L, Everett MJ, Griggs DJ (2003). Fluoroquinolone resistance in *Campylobacter* species from man and animals: detection of mutations in topoisomerase genes. J. Antimicrob. Chemother..

[42] Payot S, Cloeckaert A, Chaslus-Dancla E (2002). Selection and characterization of fluoroquinolone-resistant mutants of *Campylobacter jejuni* using enrofloxacin. Microb. Drug Resist. Epidemiol. Dis..

[43] Luo N, Sahin O, Lin J, Michel LO, Zhang Q (2003). *In vivo* selection of *Campylobacter* isolates with high levels of fluoroquinolone resistance associated with *gyrA* mutations and the function of the CmeABC efflux pump. Antimicrob. Agents Chemother..

[44] Vacher S, Ménard A, Bernard E, Mégraud F (2003). PCR-restriction fragment length polymorphism analysis for detection of point mutations associated with macrolide resistance in *Campylobacter* spp. Antimicrob. Agents Chemother..

[45] Gibreel A (2005). Macrolide resistance in *Campylobacter jejuni* and *Campylobacter coli*: Molecular mechanism and stability of the resistance phenotype. Antimicrob. Agents Chemother..

[46] Payot S (2004). Relative contribution of target gene mutation and efflux to fluoroquinolone and erythromycin resistance, in French poultry and pig isolates of *Campylobacter coli*. Int. J. Antimicrob. Agents.

[47] Hao H (2009). 23S rRNA Mutation A2074C Conferring High-Level Macrolide Resistance and Fitness Cost in *Campylobacter jejuni*. Microb. Drug Resist..

[48] Han F, Pu S, Wang F, Meng J, Ge B (2009). Fitness cost of macrolide resistance in *Campylobacter jejuni*. Int. J. Antimicrob. Agents.

[49] Ladely SR, Meinersmann RJ, Englen MD, Fedorka-Cray PJ, Harrison MA (2009). 23S rRNA gene mutations contributing to macrolide resistance in *Campylobacter jejuni* and *Campylobacter coli*. Foodborne Pathog. Dis..

[50] Maiden MCJ (1998). Multilocus sequence typing: A portable approach to the identification of clones within populations of pathogenic microorganisms. Proc. Natl. Acad. Sci..

[51] Dingle KE (2001). Multilocus sequence typing system for *Campylobacter jejuni*. J. Clin. Microbiol..

[52] The European Committee on Antimicrobial Susceptibility Testing. Breakpoint tables for interpretation of MICs and zone Version 9.0, 2019. Available at: eucast.org/clinical_breakpoints/

[53] Tewolde R (2016). MOST: a modified MLST typing tool based on short read sequencing. PeerJ.

[54] Jolley, K. A., Bray, J. E. & Maiden, M. C. J. Open-access bacterial population genomics: BIGSdb software, the PubMLST.org website and their applications [version 1; referees: 2 approved]. *Wellcome Open Res*. **3** (2018). 10.12688/wellcomeopenres.14826.1 PMC6192448 30345391

[55] Swift, C. *et al*. Comparison of phenotypic and whole genome sequencing-derived antimicrobial resistance profiles of *Campylobacter jejuni* and *C. coli* isolated from cases of diarrhoeal disease in England and Wales, 2015-2016. *J. Antimicrob. Chemother*. (2020). 10.1093/jac/dkz539 31943013

[56] Bankevich A (2012). SPAdes: A New Genome Assembly Algorithm and Its Applications to Single-Cell Sequencing. J. Comput. Biol..

[57] Treangen TJ, Ondov BD, Koren S, Phillippy AM (2014). The Harvest suite for rapid core-genome alignment and visualization of thousands of intraspecific microbial genomes. Genome Biol..

[58] Price, M. N., Dehal, P. S. & Arkin, A. P. FastTree 2 - Approximately maximum-likelihood trees for large alignments. *Plos One* **5** (2010). 10.1371/journal.pone.0009490 PMC2835736 20224823

[59] Seemann, T. Snippy-Rapid haploid variant calling and core SNP phylogeny. *GitHub*. Available at: github.com/tseemann/snippy/ (2015).

[60] Croucher NJ (2015). Rapid phylogenetic analysis of large samples of recombinant bacterial whole genome sequences using Gubbins. Nucleic Acids Res..

[61] Rambaut A, Lam TT, Max Carvalho L, Pybus OG (2016). Exploring the temporal structure of heterochronous sequences using TempEst (formerly Path-O-Gen). Virus Evol..

[62] Jolley KA, Maiden MCJ (2010). BIGSdb: Scalable analysis of bacterial genome variation at the population level. BMC Bioinformatics.

[63] Bouckaert RR (2014). BEAST 2: A Software Platform for Bayesian Evolutionary Analysis. PLoS Comput. Biol..

[64] Darriba D, Taboada GL, Doallo R, Posada D (2012). jModelTest 2: more models, new heuristics and parallel computing. Nat. Methods.

